# Defining the genomic signature of the parous breast

**DOI:** 10.1186/1755-8794-5-46

**Published:** 2012-10-11

**Authors:** Suraj Peri, Ricardo López de Cicco, Julia Santucci-Pereira, Michael Slifker, Eric A Ross, Irma H Russo, Patricia A Russo, Alan A Arslan, Ilana Belitskaya-Lévy, Anne Zeleniuch-Jacquotte, Pal Bordas, Per Lenner, Janet Åhman, Yelena Afanasyeva, Robert Johansson, Fathima Sheriff, Göran Hallmans, Paolo Toniolo, Jose Russo

**Affiliations:** 1 Department of Biostatistics and Bioinformatics, Philadelphia, PA, 19111, USA; 2 Breast Cancer Research Laboratory, Fox Chase Cancer Center, Philadelphia, PA 19111, USA; 3 Department of Obstetrics and Gynecology, New York University School of Medicine, New York, NY, 10016, USA; 4 Division of Epidemiology, New York University School of Medicine, New York, NY, 10016, USA; 5 Division of Biostatistics, Department of Environmental Medicine, New York University School of Medicine, New York, NY, 10016, USA; 6 Sunderby Hospital, Luleå and the Norrbotten Mammography Screening Program, Luleå, Sweden; 7 Departments of Radiation Sciences and Oncology, Umeå University, Umeå, Sweden; 8 Department of Public Health and Clinical Medicine, Umeå University, Umeå, Sweden; 9 Institute of Social and Preventive Medicine, Centre Hospitalier Universitaire Vaudois, Lausanne, Switzerland

**Keywords:** Gene expression profiling, Pregnancy, Breast morphology, Breast differentiation, Parous and nulliparous breast transcriptome, Breast cancer risk, Normal breast transcriptome, Bioinformatics.

## Abstract

**Background:**

It is accepted that a woman's lifetime risk of developing breast cancer after menopause is reduced by early full term pregnancy and multiparity. This phenomenon is thought to be associated with the development and differentiation of the breast during pregnancy.

**Methods:**

In order to understand the underlying molecular mechanisms of pregnancy induced breast cancer protection, we profiled and compared the transcriptomes of normal breast tissue biopsies from 71 parous (P) and 42 nulliparous (NP) healthy postmenopausal women using Affymetrix Human Genome U133 Plus 2.0 arrays. To validate the results, we performed real time PCR and immunohistochemistry.

**Results:**

We identified 305 differentially expressed probesets (208 distinct genes). Of these, 267 probesets were up- and 38 down-regulated in parous breast samples; bioinformatics analysis using gene ontology enrichment revealed that up-regulated genes in the parous breast represented biological processes involving differentiation and development, anchoring of epithelial cells to the basement membrane, hemidesmosome and cell-substrate junction assembly, mRNA and RNA metabolic processes and RNA splicing machinery. The down-regulated genes represented biological processes that comprised cell proliferation, regulation of IGF-like growth factor receptor signaling, somatic stem cell maintenance, muscle cell differentiation and apoptosis.

**Conclusions:**

This study suggests that the differentiation of the breast imprints a genomic signature that is centered in the mRNA processing reactome. These findings indicate that pregnancy may induce a safeguard mechanism at post-transcriptional level that maintains the fidelity of the transcriptional process.

## Background

Epidemiological data from various parts of the world have consistently shown that early full term pregnancy and multiparity are associated with breast cancer risk reduction in postmenopausal women [1-3], whereas late pregnancy and nulliparity are associated with increased risk [4]. It has been postulated that the mechanism of pregnancy-induced protection is mediated by changes in environmental settings [5], and/or alterations in the immunological profile of the host [6]. Animal studies of the differentiation of the breast [7-9] under the influence of the complex hormonal milieu created by two newly formed endocrine organs, the placenta and the fetus [10], have unraveled the morphological, functional, genomic and transcriptomic changes that ultimately result in the induction of a permanent and specific profile that serves as an indicator of reduced cancer risk [11,12]. There is some evidence supporting the concept that the degree of differentiation acquired through an early pregnancy changes the genomic signature that differentiates the lobular structures of parous from that of nulliparous women [3,11-18]. Our efforts have been directed towards characterizing the molecular basis underlying the mechanism of pregnancy-induced protection [3,11,12,14,18].

One way to assess whether a specific genomic fingerprint is permanently imprinted in the breast by a full term pregnancy (FTP) is to compare the transcriptomic profiles of breasts from parous and nulliparous women. We have used a genome-wide approach to identify long-term genomic changes associated with FTP by studying breast core needle biopsies (CNBs) obtained from an ethnically homogeneous population of healthy postmenopausal volunteers residing in Norrbotten County, Sweden. We previously reported on the genes differentially expressed in parous and nulliparous women using a discovery/validation approach [19]. In this paper, we describe the transcriptomic differences that were found between the breasts of parous and nulliparous women. In order to gain more statistical power in understanding the biological meaning of the transcriptomic differences, in this study the data from the discovery and validation phases were pooled and mined. Furthermore to mine the data depending on gravida status, we stratified the analyses depending on gravida status to identify importance of full-term pregnancy. Our results suggest that the differentiation of the breast induced by pregnancy imprints a genomic signature that can be detected in postmenopausal women, thus contributing to the establishment of the molecular basis of the protection against breast cancer conferred by parity.

## Methods

To determine whether the pattern of gene expression differed between nulliparous and parous postmenopausal women, breast tissue was collected from volunteering healthy women residing in Norrbotten County, Sweden, an ethnically homogeneous population of Swedish or Finnish ancestry [19]. A total of 389 women from a group who had received normal mammograms within the year prior to enrollment were initially interviewed between September 2008 and May 2009. 255 women fulfilled the eligibility criteria and signed an informed consent to participate in the study and to donate breast tissues in the form of core needle biopsies (CNB), and blood. We previously described various criteria that were used in determining the eligibility of those included in this study [19], such as women between 50 and 69 years of age, postmenopausal; i.e., lack of menstrual periods for 12 preceding months and elevated circulating levels of follicle stimulating hormone (FSH) (40–250 IU/L).

Based on reproductive history, eligible subjects were categorized as either parous or nulliparous. The parous group (P) included all women who had been pregnant (Gravida) one or more times and had delivered (parous) one or more live children. The nulliparous group (NP) included both nulligravida women who had never become pregnant and therefore never had a full term delivery, and women who had become pregnant one or more times (G≥1) but never completed a FTP, identified as nulligravida nulliparous (NN) and gravida nulliparous (GN), respectively. Both NN and GN women were considered as a single group (NP) for most analyses, unless indicated otherwise. The study protocol number 08-020M was approved by the Regional Ethical Review Board at the University of Umeå, Sweden. The study protocol number 02–829 was approved by the Institutional Review Board of Fox Chase Cancer Center, Philadelphia, USA.

### Data and sample collection

All eligible subjects signed an informed consent and completed a questionnaire that collected data on reproductive history, medical history, family background of cancer, use of tobacco, oral contraceptive (OC), hormone replacement therapy (HRT), and/or other medications.

Breast core needle biopsies (CNBs) with 14 Gauge BARD® MONOPTY® disposable core biopsy instrument (Bard Biopsy Systems, Tempe, AZ) were performed by an experienced physician at the Mammography Department at Sunderby Hospital, Luleå, Sweden. Three to five CNBs were taken free hand from the upper outer quadrant of either right or left breast; one core was fixed in 70% ethanol for histopathological analysis and the remaining cores were placed in RNAlater® (Ambion, Austin, TX) solution for subsequent RNA extraction for genomic analysis. In addition to breast tissue samples, each participant provided blood and saliva samples that were stored at Umeå University at −20°C for subsequent laboratory analyses [19].

### RNA isolation

Total RNA from CNB specimen was isolated using the Qiagen Allprep RNA/DNA Mini Kit according to manufacturer's instructions (Qiagen, Alameda, CA, USA). The quantity of total RNA obtained from every specimen ranged from 150ng to 4μg, as determined using NanoDrop v3.3.0 (NanoDrop Technologies, Wilmington, DE); RNA quality was assessed using an Agilent 2100 Bioanalyzer (Agilent Technologies, CA, USA).

### Microarray analysis

The GeneChip Expression 3’-Amplification Two-Cycle cDNA Synthesis Kit (Affymetrix, Santa Clara, CA) was used to prepare the cRNA for hybridization following the manufacturer’s protocol. The samples were hybridized to Affymetrix HG_U133 Plus 2.0 oligonucleotide arrays. 113 chips (71 parous, 42 nulliparous) satisfied quality control thresholds based on standard Affymetrix quality control measures and graphical criteria based on probe-level model (PLM) analysis as implemented in the Bioconductor affyPLM package. Affymetrix CEL files were pre-processed using RMA [20]. To account for between-batch variability in the arrays, the data were adjusted using ComBat [21]. After filtering, 18,694 probesets remained for further analysis.

**Figure 1:**
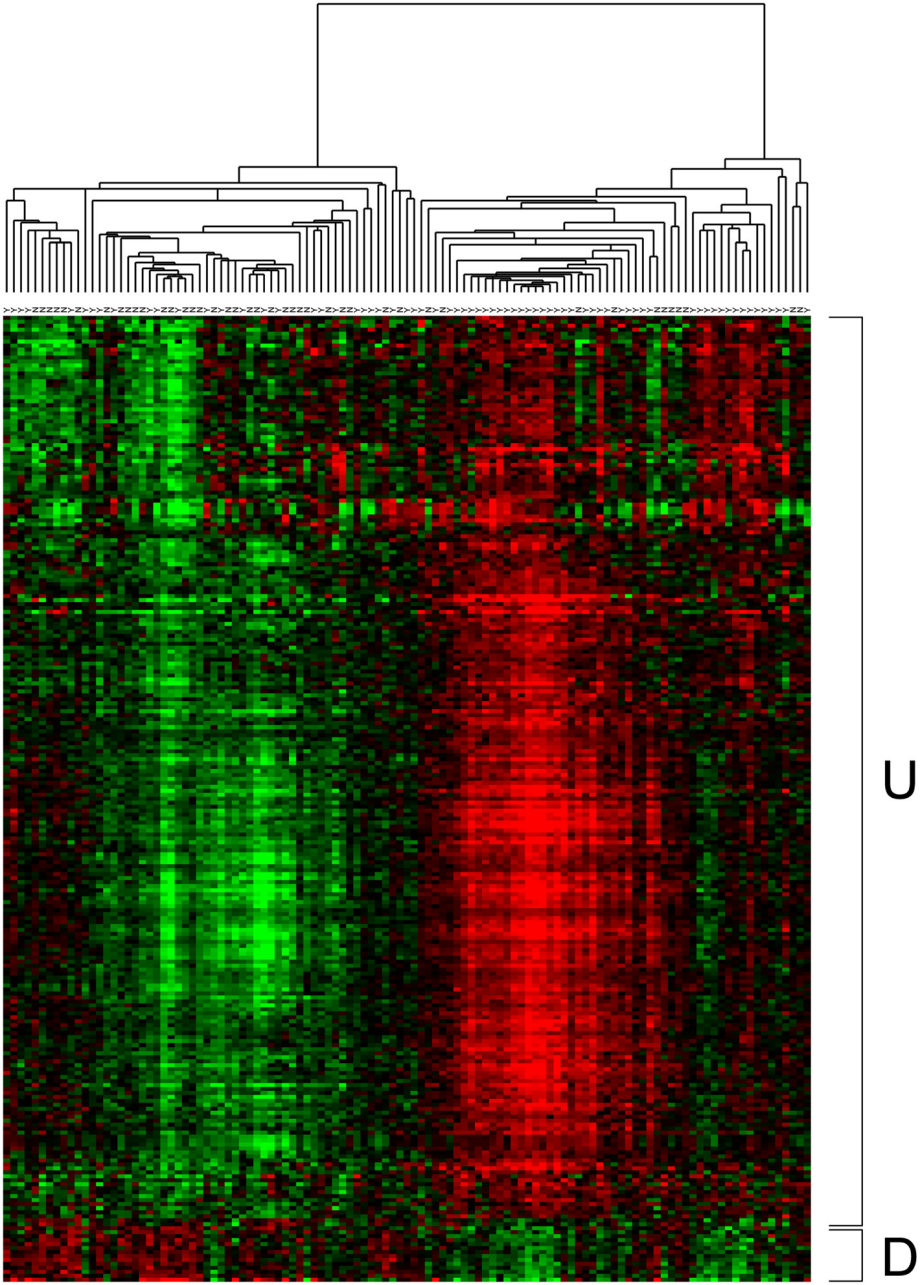
**Hierarchical clustering of differentially expressed probesets in parous and nulliparous women.** Red represents expression values above the median across all samples, and green represents values below the median. In the two top-level clusters of samples, the right cluster is composed mainly of parous samples and the left cluster is composed mainly of nulliparous samples. ‘U’ represents the intensity of up-regulated probesets among parous samples whereas ‘D’ represents the intensity of down-regulated probesets. N represents Nulliparous and Y represents Parous. A chi-square test of independence on parity status and sub-tree membership resulted in a p-value of 0.001.

To identify differentially expressed probesets, we used the limma package [22,23] implemented in the R/Bioconductor platform [24]. False Discovery Rates (FDR) were calculated using the Benjamini-Hochberg method [25]. In selecting probesets for downstream analysis, we used both a p-value of 0.001 from the empirical Bayes moderated t-statistics, and a minimum log2 fold-change of 0.3 threshold as criteria of significance, unless otherwise noted. A clustered heatmap of samples and selected genes was generated using the RMA expression values, uncentered Pearson correlation as a similarity measure and average linkage (Figure 1). The microarray data of this study have been submitted to the Gene Expression Omnibus database (GSE26457).

### Mining for functional categories and pathways

We applied data mining methods to identify enriched biological processes and pathways. Gene ontology (GO) functional categories enriched in differentially expressed genes were identified using conditional hypergeometric tests in the Bioconductor GOstats package. We carried out this analysis independently for up and down regulated genes while selecting the genes represented on U133plus2 chip as gene universe. An enrichment p-value cut off of 0.01 was used to select GO terms.

To identify pathways and associations to other previously described datasets, Gene Set Enrichment Analysis (GSEA) [26] was performed. Since we were interested in finding pathways and co-regulated genes, we expanded the list of differentially expressed genes by relaxing the p-value to 0.01 and did not apply any fold change filter. Pathways obtained from MSigDB (database of gene sets provided by GSEA) were tested for enrichment. Default parameters were chosen, except that in the case of genes with multiple probesets, only the probeset with maximum expression intensity was considered for analysis.

### Validation through real time RT-PCR

Total RNA was reverse-transcribed (RT) using MMLV reverse transcriptase (Ambion, Austin, TX) and anchored oligo-dT. Real-time Taqman PCR Assays-on-Demand were run using Universal PCR master mix (Applied Biosystems, Foster City, CA) on a 7900 HT instrument. For each gene, the log2 fold change between parous and nulliparous samples was estimated as the difference in median Ct values. To assess the statistical significance of the differences, two-sample Wilcoxon tests were performed, and comparisons with p-value <0.05 were considered statistically significant. For comparison with the microarray study, log2 fold changes were estimated as the differences in median batch-adjusted RMA normalized gene expression intensities in the same subset of 28 samples.

### Histopathological and immunohistochemical analyses

From each breast biopsy collected, one core was fixed in 70% ethanol and processed for histopathological and immunohistochemical (IHC) analyses following standard procedures. IHC analysis for cyclin L2 (CCNL2) was performed in 21 NP and 29 P CNBs tissues utilizing a polyclonal anti-human cyclin L2 (CCNL2) antibody (Novus Biologicals, Cambridge, UK) at a concentration of 5μg/ml. Reactions were performed using the MultiLink Detection Kit for HRP/DAB and the i6000 automatic stainer (both from Biogenex, San Ramon, CA). CCNL2 positive cells were scored according to the intensity of brown nuclear stain as negative (0), weakly positive (+) or strongly positive (++). Results were expressed as the percentage of positive cells over total number of epithelial cells present in ducts and lobules type 1 in each section.

## Results

### Volunteers included in the analysis

As described in Methods, the study participants consisted of postmenopausal women that were grouped according to their reproductive history into parous (P) and nulliparous (NP). The nulliparous group included both, nulligravida nulliparous (NN) and gravida nulliparous (GN); both NN and GN women were considered within the NP as a single group for most analyses, unless indicated otherwise. CNBs were first analyzed histopathologically in order to determine the adequacy of tissue, presence of ductal and lobular structures and the characteristics of the stroma. A total of 126 biopsies obtained from 82 P and 44 NP women were eligible for final genomic analysis. The group of nulliparous women was 58.9±5.2 years old and the group of parous women was 59.6±5.8 years old and there were not statistic differences among the two groups. Therefore a variation in age subsets was not detected in this study, and altogether the data reflect a well controlled group of postmenopausal women.

### Genomic analysis

From the 126 samples hybridized to Affymetrix HG_U133 Plus 2.0 oligonucleotide arrays, 113 chips (71 P, 42 NP) satisfied quality control thresholds. Using empirical Bayes moderated t-statistics with p-value less than 0.001 and a minimum log2 fold-change of 0.3 thresholds as criteria of significance, we identified 305 differentially expressed probesets (corresponding to 208 distinct genes) between P and NP women (see Additional file 1: Table S1). Of these, 267 were up-regulated and 38 were down-regulated (Figure 1). In order to test the validity of the list of differentially expressed genes, the parity status labels were randomly permuted and the analysis was repeated 10,000 times. Over half of the permutations yielded either 0 or 1 differentially expressed gene, the 95th percentile of counts was 15 differentially expressed genes, and only 8 out of 10,000 permutations yielded at least 305 differentially expressed genes (i.e. p-value = 0.0008) suggesting the differentially expressed gene signature does not arise by chance. Hierarchical clustering of the differentially expressed probesets (with no sample clustering) shows the pattern of up and down regulated genes within each group (see Additional file 1: Figure S1). To understand the biological theme of the observed gene expression differences, we carried out bioinformatics-based analysis of microarray data.

Gene ontology (GO) enrichment analysis revealed biological processes that were categorized into groups including RNA metabolic processes, differentiation and development of epidermis and ectoderm, and cell-substrate junction assembly (Table 1), findings that are in agreement with existing knowledge that pregnancy hormones promote the differentiation of mammary epithelial cells [3]. Highly represented in the parous breast were biological processes involving both mRNA and RNA metabolic processes and RNA splicing machinery. Important genes that were up-regulated within these categories were: RBMX, HNRNPA1, HNRNPA2B1, HNRNPD, LUC7L3, PNN, PRPF39, RBM25, SFPQ, SFRS1, SFRS5, SFRS7, PABPN1, and PRPF4B. Biological processes such as differentiation and development of epithelial and ectodermal cells were represented by the up-regulation of COL7A1, KRT5, KRT15, LAMA3, LAMC2, NTF4, and KLK7. We also found that genes which are pivotal in two biological processes that are critical to the anchoring of epithelial cells to the basement membrane, hemidesmosome and cell-substrate junction assembly, such as KRT5, LAMA3 and LAMC2, were up-regulated in the P group (Table 1).

**Table 1:** GO biological processes enriched for both up and down regulated genes between parous and nulliparous breast samples

**GO term ID**	**GO term (p-value)**	**Genes up-regulated in parous samples**
GO:0007044	cell-substrate junction assembly (0.006)	KRT5, LAMA3, LAMC2
GO:0007398	ectoderm development (0.002)	COL7A1, KRT5, KRT15, LAMA3, LAMC2, NTF4, KLK7
GO:0008544	epidermis development (0.001)	COL7A1, KRT5, KRT15, LAMA3, LAMC2, NTF4, KLK7
GO:0010160	formation of organ boundary(0.009)	NTF4
GO:0031581	hemidesmosome assembly (0.000)	KRT5, LAMA3, LAMC2
GO:0016071	mRNA metabolic process (0.000)	CIRBP, RBMX, HNRNPA1, HNRNPA2B1, HNRNPD, LUC7L3, PNN, PRPF39, RBM25, SFPQ, SFRS1, SFRS5, SFRS7, PABPN1, PRPF4B
GO:0006397	mRNA processing (0.000)	RBMX, HNRNPA1, HNRNPA2B1, LUC7L3, PNN, PRPF39, RBM25, SFPQ, SFRS1, SFRS5, SFRS7, PABPN1, PRPF4B
GO:0034059	response to anoxia (0.009)	OXTR
GO:0016070	RNA metabolic process (0.006)	DDX17, CHD2,C BX3, CIRBP, ZNF785, EZH2, L3MBTL, GATA3, RBMX, ZNF789, HNRNPA1, HNRNPA2B1, HNRNPD, LUC7L3, PNN, PRPF39, ZNF83, METTL3, CREBZF, RBM25, RBBP8, RPS24, CENPK, SFPQ, SFRS1, SFRS5, SFRS7, ZNF814, ZNF207, PABPN1, RUNX3, FUBP1, PRPF4B, HNRPDL
GO:0006396	RNA processing (0.000)	DDX17, RBMX, HNRNPA1, HNRNPA2B1, HNRNPD, LUC7L3, PNN, PRPF39, RBM25, RPS24, SFPQ, SFRS1, SFRS5, SFRS7, PABPN1, PRPF4B, HNRPDL
GO:0008380	RNA splicing (0.000)	RBMX, HNRNPA1, HNRNPA2B1, HNRNPD, LUC7L3, PNN, PRPF39, RBM25, SFPQ, SFRS1, SFRS5, SFRS7, PABPN1, PRPF4B
**GO term ID**	**GO term**	**Genes down-regulated in parous samples**
GO:0032859	activation of Ral GTPase activity (0.003)	RALGAPA2
GO:0021534	cell proliferation in hindbrain (0.005)	IGF1
GO:0009441	glycolate metabolic process (0.001)	IGF1
GO:0042692	muscle cell differentiation (0.006)	IGF1, SOX6
GO:0051450	myoblast proliferation (0.003)	IGF1
GO:0006654	phosphatidic acid biosynthetic process (0.005)	ABHD5
GO:0031325	positive regulation of cellular metabolic process (0.005)	EBF1, IGF1, ABHD5, SOX6
GO:0045821	positive regulation of glycolysis (0.006)	IGF1
GO:0051006	positive regulation of lipoprotein lipase activity (0.006)	ABHD5
GO:0042523	positive regulation of tyrosine phosphorylation of Stat5 protein (0.009)	IGF1
GO:0031329	regulation of cellular catabolic process (0.002)	IGF1, ABHD5
GO:0043567	regulation of insulin-like growth factor receptor signaling pathway (0.007)	IGF1
GO:0010660	regulation of muscle cell apoptosis (0.006)	IGF1
GO:0032485	regulation of Ral protein signal transduction (0.003)	RALGAPA2
GO:0010889	regulation of sequestering of triglyceride (0.006)	ABHD5
GO:0033143	regulation of steroid hormone receptor signaling pathway (0.010)	IGF1
GO:0090207	regulation of triglyceride metabolic process (0.008)	ABHD5
GO:0043403	skeletal muscle tissue regeneration (0.009)	IGF1
GO:0007264	small GTPase mediated signal transduction (0.005)	IGF1, RASD1, RALGAPA2
GO:0035019	somatic stem cell maintenance (0.010)	IGF1

Among the down-regulated genes, insulin-like growth factor 1 (IGF-1) was enriched in 19 biological processes that comprised cell proliferation, regulation of IGF-like growth factor receptor signaling, somatic stem cell maintenance, muscle cell differentiation and apoptosis, among others. Other down-regulated genes were RALGAPA2, SOX6, ABHD5, EBF1 and RASD1 (Table 1).

We used gene set enrichment analysis (GSEA) to compare differentially expressed genes from this study to 3717 curated gene sets of specific pathways, processes and profiles of previous profiling experiments obtained through MsigDB [26]. Pathways enriched by up-regulated genes included breast cancer estrogen signaling, cell communication and mRNA processing (Table 2). The breast cancer estrogen signaling pathway encompassed a set of genes that were dysregulated in estrogen receptor dependent breast cancers. Among these genes were SCGB2A1, SCGB2A2, GATA3, TP53, TFF1, STC2, SERPINB5 and SERPINA3. Since full-term pregnancy involves the influx of several hormones including estrogen, we postulated that several down-stream targets of estrogen would be co-regulated in parous subjects. Other pathways that were enriched by up-regulated genes were cell communication (DSC3 and KRT5) and the mRNA processing reactome (Table 2). Of great interest was the significant number of genes related to the mRNA processing reactome that were differentially expressed by parity. This pathway was comprised of those genes involved in key molecular mechanisms that encompass mRNA and pre-mRNA processing reactions, as well as splicing of mRNAs, whose representative genes include METTL3, HNRPD, HNRPA2B1, PABPN1, PRPF4B, SRSF7, CLK4, and SFRS5. Among the key pathways that were enriched by down-regulated genes the most significant ones were the insulin signaling pathway, MAPK, cytokine-cytokine receptor interaction and Wnt signaling pathways (Table 2).

**Table 2:** Enriched GSEA pathways and gene sets for both up- and down-regulated genes. ‘NES’ represents normalized enrichment score

**Pathways and gene sets enriched by up-regulated genes**	**NES**	**FDR q-val**	**Genes**
Breast cancer estrogen signaling	2.217	0.002	SCGB2A1, TFF1, SCGB2A2, SCGB1D2, STC2, GATA3, SERPINB5, SERPINA3, AZGP1, TP53, CCNA2, CCNE2, EGFR, PTEN, DLC1, FHL5
HSA01430 cell communication	1.711	0.075	KRT15, DSC3, DSG3, KRT5, COL4A6, KRT17, KRT14, LAMC2, LAMA3, LAMB3, THBS3, LAMA5,LAMC1, GJA4, LAMA4, VWF, COL4A1, COL4A2
MRNA processing reactome	1.626	0.093	METTL3, HNRPD, HNRPA2B1, PRPF4B, SFRS7, CLK4, SFRS5, PABPN1, CSTF3, HNRPU, RBM5, SNRP70, SFRS14, SNRPA1, CLK2, NXF1, SFRS8, SFRS2, PTBP2, FUS, SFRS6, SFRS16, SF3B1, HNRPA3, SNRPB, PRPF3, SFRS12, U2AF1, PHF5A, TXNL4A, CUGBP2
**Pathways and gene sets enriched by down-regulated genes**	**NES**	**FDR q-val**	**Genes**
HSA04910 insulin signaling pathway	−2.242	0.004	CALML3, SHC4, IKBKB, PKM2, PIK3CD, PHKA1, CALM1, CBL, MAPK9, GSK3B, SKIP, MAP2K1, PIK3R3, CRK, IRS2, SORBS1, SOS1, PDE3A, PDE3B, PRKAR2B, MAPK10, PCK1
HSA04080 neuroactive ligand receptor interaction	−2.148	0.007	NPY2R, OXTR, PARD3, GABBR1, LTB4R, NR3C1, EDNRA, EDNRB, EDG1, CALCRL, ADRB2, AGTRL1, ADRA2A, GHR, PTGER3, ADRA1A
HSA04530 tight junction	−2.053	0.012	CGN, INADL, EXOC3, LLGL2, PARD3, PARD6G, TJP1, EPB41L1, ZAK, PPP2R1B, PTEN, GNAI1, RRAS2, CTNNA1, MAGI1, CLDN10
HSA04010 MAPK signaling pathway	−2.018	0.013	NTF5, FGFR3, CACNA1D, RASGRP1, MAP3K14, TP53, CACNA1G, FAS, PDGFA, IKBKB, CACNA2D2, DAXX, GNA12, MAPK9, EGFR, GADD45A, DUSP10, CHP, DUSP3, MAP2K1, CRK, MEF2C, ZAK, EVI1, TGFBR1, SOS1, FGF10, RAPGEF2, RRAS2, RASGRF2, MAPK10, ACVR1C
HSA05210 colorectal cancer	−1.972	0.013	TP53, IGF1R, FZD8, RALGDS, PIK3CD, FZD6, CYCS, MAPK9, EGFR, GSK3B, MAP2K1, PIK3R3, TGFBR1, SOS1, TCF7L2, FZD4, MAPK10, ACVR1C
HSA04510 focal adhesion	−1.913	0.016	PAK7, COL4A6, LAMC2, LAMA3, SHC4, PAK6, ITGA10, LAMB3, ITGA4, PDGFA, IGF1R, THBS3, LAMA5, ZYX, PPP1R12A, ITGA2, PIK3CD, MAPK9, CAV1, EGFR, PARVA, GSK3B, LAMC1, MAP2K1, PIK3R3, CRK, FLT1, FYN, CCND2, PTEN, LAMA4, SOS1, VWF, IGF1, CAV2, TLN2, COL4A1, PDGFC, COL4A2, MAPK10
Integrin mediated cell adhesion KEGG	−1.661	0.062	CAPN3, PAK6, ITGA10, ITGA4, ZYX, ITGA2, CAV1, MAP2K1, CRK, SORBS1, FYN, SOS1, CAV2, TNS1, MAPK10
HSA04310 WNT signaling pathway	−1.423	0.161	CSNK1A1, NFATC3, TP53, WNT5A, VANGL2, FZD8, FZD6, MAPK9, GSK3B, CHP, DAAM1, DAAM2, SOX17, PPP2R1B, CCND2, TCF7L2, FZD4, MAPK10
ST INTEGRIN SIGNALING PATHWAY	−1.400	0.160	PAK7, PAK6, ITGA10, ITGA4, ZYX, ITGA2, MAPK9, CAV1, CRK, ARHGEF7, FYN, PTEN, SOS1, TLN2, MAPK10
HSA04060 cytokine cytokine receptor interaction	−1.383	0.152	CXCL6, IL28RA, CCL5, IFNGR2, FAS, IL4R, TNFSF15, PF4V1, TNFRSF14, IL2RB, IFNAR1, EGFR, LIFR, FLT1, BMPR2, TGFBR1, PDGFC, GHR, BMP2.

### Contribution of full-term pregnancy (FTP) to transcriptomic changes

To investigate whether an incomplete pregnancy could induce transcriptomic changes in the breast tissue, in the nulliparous group (NP) we compared gene expression of GN against NN. We did not find any significant differences in gene expression between these two subgroups. Comparison between P and GN revealed that 12 genes (18 probes) were differentially expressed (see Additional file 1: Table S2). The comparison between P and NN revealed that 125 genes (206 probes) (see Additional file 1: Table S3) were differentially expressed, and among these, 107 genes had been identified in the comparison P vs. NP. These results suggest that in this study population, FTP was required for inducing detectable changes in the transcriptome.

### Validation of microarray results

**Figure 2:**
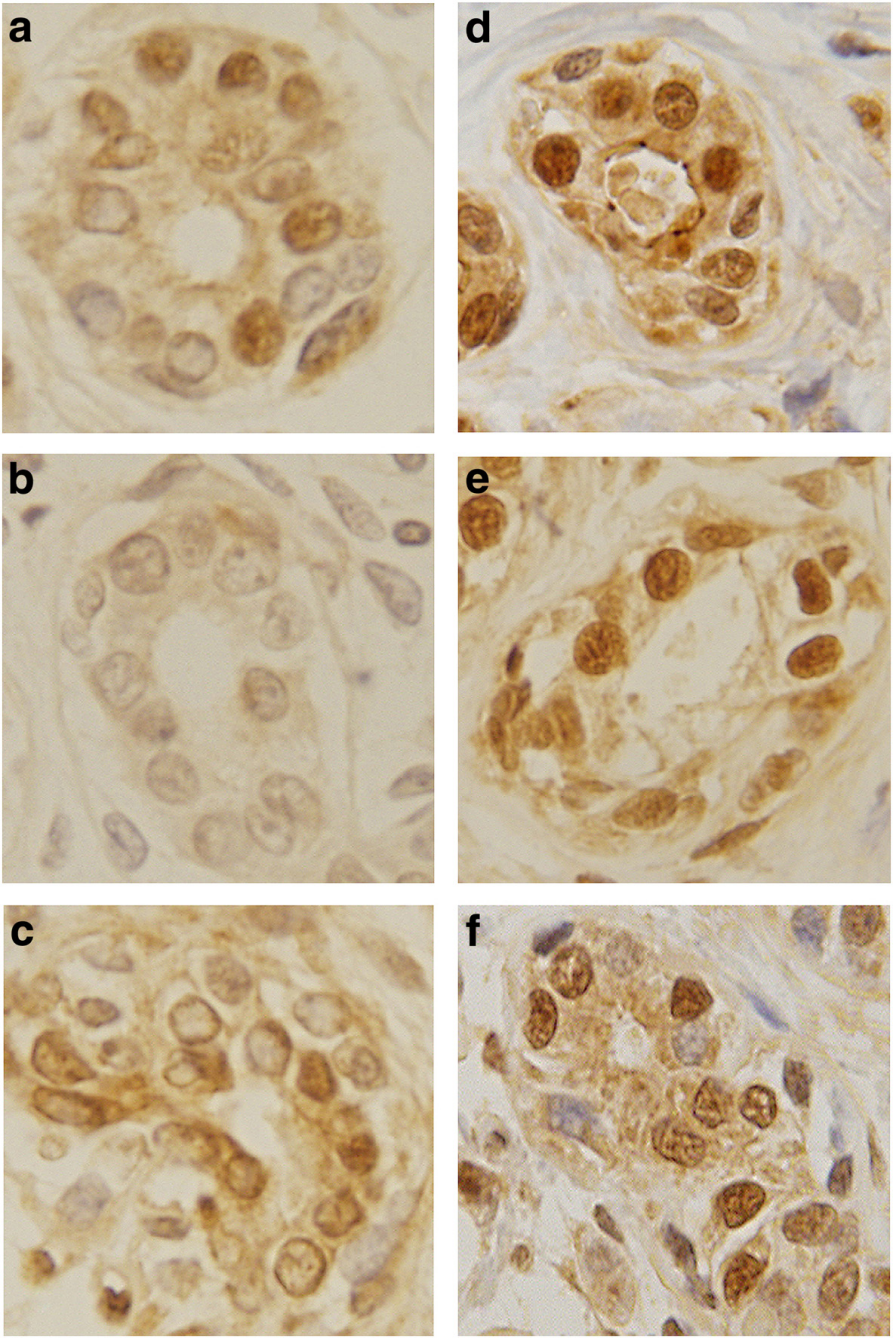
**Immunohistochemistry of cyclin-cyclin L2 protein (CCNL2) performed in paraffin embedded tissues of nulliparous and parous samples.** CCNL2 protein was overexpressed in the nucleus of epithelial cells of lobules type 1 in parous breast (**d**,**e**,**f**) when compared to nulliparous women (**a**,**b**,**c**) (40X).

Due to limitation in availability of RNA required for gene expression validation, the following genes were selected based on statistical significance of differentially expressed genes and their biological relevance: XIST, CREBZF, CCNL2, AHSA2, CIRBP, PILRB, OXTR, TNMD and SOX6 (Table 3). These genes displayed the same expression behavior in both microarray and real time RT-PCR. XIST, CREBZF and CCNL2 were significantly (p<0.05) up-regulated in the parous women. In addition, the level of expression and localization of CCNL2 was verified by immunohistochemistry in nulliparous and parous breasts (Figure 2). CCNL2 protein was significantly overexpressed in the nucleus of epithelial cells of lobules type 1 of the parous breast (Figure 2 d,e,f) when compared with similar structures found in the breast of nulliparous women (Figure 2 a,b,c). These observations confirm the localization of this protein in the splicing factor compartment (nuclear speckles) [27].

**Table 3:** RT-PCR validation results

**ABI assay**	**Gene symbol**	**Gene name**	**Log ratio**	**p-value**	**95% Cl**
Hs00221881_m1	CREBZF	CREB/ATF bZIP transcription factor	1.59	0.000	[0.70, 2.54]
Hs00300535_s1	XIST	X (inactive)-specific transcript (non-protein coding)	1.19	0.006	[0.37, 1.95]
Hs01085988_m1	CCNL2	Cyclin L2	0.75	0.030	[0.05, 1.42]
Hs00902302_m1	AHSA2	AHA1, activator of heat shock 90kDa protein ATPase homolog 2 (yeast)	0.78	0.002	[0.34, 1.87]
Hs00154457_m1	CIRBP	Cold inducible RNA binding protein	0.46	0.019	[0.06, 0.94]
Hs00273801_m1	PILRB	Paired immunoglobin-like type 2 receptor beta	1.86	0.002	[0.72, 3.11]
Hs00168573_m1	OXTR	Oxytocin receptor	1.99	0.002	[0.89, 2.74]
Hs00223332_m1	TNMD	Tenomodulin	−1.27	0.012	[−2.52, -0.11]
Hs00264525_m1	SOX6	SRY (sex determining region Y)-box 6	−0.59	0.118	[−1.215, 0.13]

## Discussion

The work reported here demonstrates that differentiation of the breast induced by an early pregnancy imprints a specific genomic signature that can be detected in postmenopausal women. Using bioinformatics methods we found transcriptomic differences between the breasts of parous and nulliparous women. These differentially expressed genes were used to identify enriched biological processes and pathways. Enriched biological processes related to up-regulated genes included RNA related processes, differentiation and development of epidermis and ectoderm, and cell-substrate junction assembly; whereas in the case of down-regulated genes the biological processes that were enriched included IGF-like growth factor signaling, somatic stem cell maintenance and apoptosis. Pathways that were enriched by up-regulated genes included breast cancer estrogen signaling, cell communication and mRNA processing machinery. Numerous pathways were enriched by down-regulated genes; the most significant ones were the insulin, Wnt and integrins signaling pathways, MAPK, cytokine-cytokine receptor interaction, tight junction and focal adhesion, all representing proteins that are highly expressed in malignancies.

The main components of the spliceosome machinery, including RNA and proteins that undergo dynamic changes during the splicing reaction, were up-regulated in the parous breast. Among them were the heterogeneous nuclear ribonucleoproteins (HNRPs) that include HNRPA3, HNRPA2B1, HNRPD and HNRPU [28], which are implicated in the regulation of mRNA stability, as well as other functions, such as mammary gland involution [29], negative regulation of telomere length maintenance [30], and regulation of mRNA trafficking from the nucleus to distal processes in neural cells [31]. Although further studies are needed to define their precise functional role in the postmenopausal breast, we postulate that they may play an important regulatory function as transcriptional regulators. In addition, post-transcriptional methylation of internal adenosine residues in eukaryotic mRNAs by METTL3 (methyltransferase like 3), which is up-regulated in the parous breast, could play a role in the efficiency of mRNA splicing, transport or translation in the differentiated breast epithelium. Other members of the spliceosome complex are the proteins encoded by the genes SF3B1, SFRS2, SFRS7, SFRS8, SFRS14, SFRS16, SNRP70, SNRPB, SNRPA1, PRF3 and PHF5A, all of which are overexpressed in the parous breast. In the case of the small nuclear ribonucleoproteins (snRNPs), there is evidence that they suppress tumor cell growth and may have major implications as cancer therapeutic targets. The pre-mRNA splicing factors are enriched in nuclear domains termed interchromatin granule clusters or nuclear speckles. Among the members of the splicing factor compartment are CCNL1 and CCNL2 that participate in the pre-mRNA splicing process and are located in the nuclear speckles [32,33]. These two genes are up-regulated in the parous breast and the CCNL2 protein is also overexpressed in the nucleus of breast epithelial cells. CCNL1 and CCNL2 are transcriptional regulators [32,33] that modulate the expression of critical factors leading to cell apoptosis, possibly through the Wnt signal transduction pathway [34], a signaling pathway that is enriched by down-regulated genes in the parous breast (Table 2). In our previously published preclinical and clinical studies [3,7,9,11], we have reported that pregnancy confers protection from breast cancer development by inducing gland differentiation, which imprints a specific and permanent genomic signature in this organ. A similar phenomenon was demonstrated in the breast of postmenopausal parous women characterized by fatty involution [3]. We previously described a small case–control study of transcriptomic analysis of normal breast tissues obtained from parous and nulliparous women free of breast pathology and parous and nulliparous women with history of breast cancer that served as controls and cases, respectively [3]. In order to investigate the degree of commonality between the previous case–control and the present study, we applied GO enrichment analysis to the gene lists generated from both studies. We found that processes involved in RNA metabolism and RNA processing were similar in both studies (see Additional file 1: Table S4).

A number of non-coding RNAs that included XIST, MALAT-1 (also called NEAT2) and NEAT1 were up-regulated in the parous breast. XIST, which inactivates X chromosome as an early developmental process, plays an essential role in female mammals by providing dosage equivalence between males and females. Up-regulation of XIST occurs upon differentiation, whereas failure to express XIST is often seen in malignancies and in early embryogenesis [35]. Our findings are supported by recent reports that suggest that XIST is expressed in adult well-differentiated cells in order to maintain gene repression [35-39]. Oxytocin, a neurotransmitter that acts through it specific receptor OXTR and is overexpressed during lactation, up-regulates the expression of MALAT-1, a highly conserved non-coding RNA [40,41]. Interestingly, both MALAT1 and OXTR remain overexpressed in the breast of postmenopausal parous women. NEAT1 and NEAT2 localize to the periphery and to the interior the spliceosome assembly factor SC35 domains or speckles. Our observation that in breast epithelial cells CCNL2 is highly enriched in nuclear speckles (Figure 2) indicates that CCNL2 might colocalize with NEAT1 and NEAT2. The down-regulation of NEAT1, NEAT2 and XIST in the breast of nulliparous women, in whom this organ never reached a stage of complete differentiation similar to that achieved after completion of pregnancy and lactation [42], suggests that the undifferentiated breast is not actively involved in the RNA metabolism that is necessary for maintaining a state of differentiation.

Although in this study we did not observe differential expression in estrogen receptor between parous and nulliparous breasts, several genes that are directly or indirectly regulated by estrogen receptor were up- or down-regulated in the parous breast and were found to be enriched in the breast cancer estrogen signaling gene set. Among them, GATA3, an important component of this gene set, is crucial to mammary gland morphogenesis and differentiation of progenitor cells. GATA3 has been suggested to be a tumor suppressor [43], a fact supported by the observations that induction of its expression in GATA3-negative undifferentiated carcinoma cells is sufficient to induce tumor differentiation and inhibition of tumor dissemination [44-46]. The down-regulation of RASD1 (RAS, dexamethasone-induced 1), a potential miR-375 target that negatively regulates ER alpha expression in breast cancer further confirms that the genes involved in the estrogen receptor regulated pathways could be under permanent transcriptional modification as a manifestation of a higher degree of cell differentiation of the parous breast, in spite of the lack of transcriptomic differences in the levels of the receptor between parous and nulliparous breast tissues.

Cell communication, which is a key element in the process of cell and organ differentiation, is well represented in the breast of parous women. The parous breast exhibits up-regulation of desmocollin (DSC3), a calcium-dependent glycoprotein that is a member of the desmocollin subfamily of the cadherin superfamily. Members of this desmosomal family, along with the desmogleins, are found primarily in epithelial cells where they constitute the adhesive proteins of the desmosome cell-cell junction and are required for cell adhesion and desmosome formation. In addition, the up-regulation of matrix Gla proteins (MGP), laminins (LAMA3 and LAMC2) and keratin 5 (KRT5) in the parous breast reflect the greater differentiated state of the breast epithelial cells [47]. This concept is supported by the observation that the loss of Matrix Gla protein expression may be associated with tumor progression and metastasis [48].

Our findings that insulin-like growth factor 1 (IGF-1) is down-regulated in the parous breast is consistent with published data reporting overall lower levels of IGF-1 in parous than in nulliparous women [49] and support the association of IGF1 with increased breast cancer risk [50]. It is known that IGF-1 stimulates mitosis and inhibits apoptosis, playing a significant role in signaling pathways involved in the pathogenesis of breast cancer. The down regulation of IGF-1 in the parous breast, in association with the significant down-regulation of SOX6, EBF1 (early B-cell factor 1), ABHD5, RASD1, a potential miR-375 target that negatively regulates ER alpha expression in breast cancer [51], and RALGAPA2, could represent a significant driving force in the reduction of breast cancer risk conferred by pregnancy.

## Conclusions

In this study using a core needle biopsy of postmenopausal breast parenchyma comprising of stroma and lobular structures, we found a specific genomic signature induced by FTP. This genomic signature suggests that the differentiation process of breast cells is centered in the mRNA processing reactome, which emerges as an important regulatory pathway induced by pregnancy. The biological importance of the differential expression of genes that control the spliceosome could be an indication of a safeguard mechanism at post-transcriptional level that maintains the fidelity of the transcriptional process. In addition, the critical regulatory pre-mRNA splicing mechanism could also regulate the expression of specific genes controlling estrogen signaling pathways, cell communication and differentiation, as well as pathways related to chromatin remodeling, altogether resulting in control of cell differentiation and breast cancer prevention. Future studies are needed to confirm these results, in particular studies focusing specifically on lobular epithelial cells selected using laser capture microdissection (LCM). Finally, digital transcriptome analysis such RNA-Seq methods will help in understanding the precise differentiation paradigms in parous breast tissue.

## Misc

Suraj Peri, Ricardo López de Cicco, Julia Santucci-Pereira, Michael Slifker, Eric A. Ross contributed equally to this work.

## Competing interests

The authors declare that they have no competing interests.

## Authors’ contributions

SP, MS, JS-P, ER and JR performed data analysis, interpreted the results and drafted the manuscript. RLC, JS-P, FS, PAR, IHR and JR carried out the processing of biopsy tissue, microscopy work, immunohistochemistry, expression profiling and validation experiments. AAA, IB-L,YA, AZ-J and PT designed questionnaire, managed data, performed demographic data analysis and statistical analyses. JA, PB, RJ, GH and PL implemented Swedish cohort recruitment, ethical, clinical and radiological evaluation, and data management and planning. JA and PB carried out biopsy work. PL supervised Umea University team, PT supervised NYU team, and JR supervised FCCC team. All authors read and approved the final manuscript.

## Pre-publication history

The pre-publication history for this paper can be accessed here:

http://www.biomedcentral.com/1755-8794/5/46/prepub

## Supplementary Material

Additional file 1 **Table S1.** Probesets differentially expressed in Parous versus Nulliparous (p<0.001 and log2 fold change of at least 0.3). Table S2- Genes differentially expressed by full term pregnancy (P) when compared to women that did not have a full term pregnancy (GN) (p<0.001 and log2 fold change of at least 0.3). Table S3. Genes differentially expressed in Parous (P) versus Nulligravidas (NG) (p<0.001 and log2 fold change of at least 0.3). Table S4. Comparison between biological processes that are over-represented in the two studies. Figure S1- Hierarchical clustering of differentially expressed probesets in parous and nulliparous women (samples were not clustered). Red represents expression values above the median across all samples, and green represents values below the median. The left portion of the figure is composed by nulliparous (NP) samples and the right portion is composed by parous (P) samples. ‘U’ represents the intensity of up-regulated probesets among parous samples whereas ‘D’ represents the intensity of down-regulated probesets. Click here for file
