# Supplementary material for: Defining the genomic signature of the parous breast
Source: BMC Med Genomics. 2012 Oct 11;5:46. doi: 10.1186/1755-8794-5-46 (PMC3487939; doi:10.1186/1755-8794-5-46)
Supplement: Additional file 1 — Table S1. Probesets differentially expressed in Parous versus Nulliparous (p<0.001 and log2 fold change of at least 0.3). Table S2- Genes differentially expressed by full term pregnancy (P) when compared to women that did not have a full term pregnancy (GN) (p<0.001 and log2 fold change of at least 0.3). Table S3. Genes differentially expressed in Parous (P) versus Nulligravidas (NG) (p<0.001 and log2 fold change of at least 0.3). Table S4. Comparison between biological processes that are over-represented in the two studies. Figure S1- Hierarchical clustering of differentially expressed probesets in parous and nulliparous women (samples were not clustered). Red represents expression values above the median across all samples, and green represents values below the median. The left portion of the figure is composed by nulliparous (NP) samples and the right portion is composed by parous (P) samples. ‘U’ represents the intensity of up-regulated probesets among parous samples whereas ‘D’ represents the intensity of down-regulated probesets. [file 1755-8794-5-46-S1.pdf]

Table S1- Probesets differentially expressed in Parous versus Nulliparous (p<0.001 and $\log_2$ fold change of at least 0.3)

Gene Symbol	Probe ID	Log Ratio	P- value	FDR	Gene Name
ABAT	209460_at	0.31	0.0005	0.02	4-aminobutyrate aminotransferase
ABAT	209459_s_at	0.34	0.0001	0.01	4-aminobutyrate aminotransferase
ABHD5	213805_at	-0.32	0.0003	0.01	abhydrolase domain containing 5
AFG3L1	1552287_s_at	0.35	0.0002	0.01	AFG3 ATPase family gene 3-like 1 (S. cerevisiae)
AGBL3	232395_x_at	0.48	0.0005	0.02	ATP/GTP binding protein-like 3
AHSA2	230148_at	0.42	0.0006	0.02	AHA1, activator of heat shock 90kDa protein ATPase homolog 2 (yeast)
AHSA2	226334_s_at	0.44	0.0007	0.02	AHA1, activator of heat shock 90kDa protein ATPase homolog 2 (yeast)
AHSA2	226665_at	0.65	0.0003	0.01	AHA1, activator of heat shock 90kDa protein ATPase homolog 2 (yeast)
ANKRD10	223251_s_at	0.32	0.0001	0.01	ankyrin repeat domain 10
ANXA9	211712_s_at	0.37	0.0003	0.01	annexin A9
AP1G2	201613_s_at	0.33	0.0001	0.01	adaptor-related protein complex 1, gamma 2 subunit
ARGLU1	228477_at	0.33	0.0001	0.01	arginine and glutamate rich 1
ARGLU1	218067_s_at	0.34	0.0002	0.01	arginine and glutamate rich 1
ARHGAP8	232567_at	0.33	0.0008	0.02	Rho GTPase activating protein 8
ATHL1	219359_at	0.35	0.0006	0.02	ATH1, acid trehalase-like 1 (yeast)
BZRAP1	205839_s_at	0.33	0.0000	0.01	benzodiazapine receptor (peripheral) associated protein 1
C11orf31	228331_at	0.49	0.0000	0.01	chromosome 11 open reading frame 31
C1orf168	238625_at	0.80	0.0000	0.01	chromosome 1 open reading frame 168
C1orf63	209007_s_at	0.51	0.0010	0.02	chromosome 1 open reading frame 63
C1orf63	209006_s_at	0.52	0.0006	0.02	chromosome 1 open reading frame 63
C2orf63	228316_at	0.31	0.0004	0.01	chromosome 2 open reading frame 63
C3orf15	236222_at	0.30	0.0003	0.01	chromosome 3 open reading frame 15
C4orf19	235350_at	-0.32	0.0005	0.02	chromosome 4 open reading frame 19
C6orf170	232038_at	0.38	0.0001	0.01	chromosome 6 open reading frame 170
C8orf79	239297_at	0.32	0.0002	0.01	chromosome 8 open reading frame 79
C8orf84	235209_at	0.40	0.0008	0.02	chromosome 8 open reading frame 84
CAPN3	210944_s_at	0.40	0.0006	0.02	calpain 3, (p94)
CAPN8	229030_at	0.55	0.0000	0.01	calpain 8
CASP4	213596_at	0.37	0.0003	0.01	caspase 4, apoptosis-related cysteine peptidase
CBX3	1555920_at	0.53	0.0003	0.01	chromobox homolog 3 (HP1 gamma homolog, Drosophila)
CCDC14	225017_at	0.35	0.0002	0.01	coiled-coil domain containing 14
CCDC45	225705_at	0.32	0.0002	0.01	coiled-coil domain containing 45
CCNL1	1555411_a_at	0.35	0.0009	0.02	cyclin L1
CCNL1	220046_s_at	0.40	0.0001	0.01	cyclin L2
CCNL2	222999_s_at	0.47	0.0002	0.01	cyclin L2
CD69	209795_at	0.32	0.0006	0.02	CD69 molecule
CDCA7	224428_s_at	0.31	0.0005	0.02	cell division cycle associated 7
CDK12	213557_at	0.37	0.0001	0.01	cyclin-dependent kinase 12
CDK5RAP3	218740_s_at	0.31	0.0000	0.00	CDK5 regulatory subunit associated protein 3
CENPK	222848_at	0.34	0.0001	0.01	centromere protein K
CGN	223233_s_at	0.35	0.0004	0.01	cingulin
CGN	223232_s_at	0.41	0.0002	0.01	cingulin
CHD2	228999_at	0.33	0.0001	0.01	chromodomain helicase DNA binding protein 2
CIRBP	230142_s_at	0.45	0.0000	0.01	cold inducible RNA binding protein
CIRBP	225191_at	0.55	0.0002	0.01	cold inducible RNA binding protein

CLGN	205830_at	0.47	0.0003	0.01	calmegin
CLK4	210346_s_at	0.36	0.0004	0.02	CDC-like kinase 4
CMYA5	233520_s_at	0.46	0.0000	0.01	cardiomyopathy associated 5
COL16A1	204345_at	0.31	0.0000	0.01	collagen, type XVI, alpha 1
COL27A1	225293_at	0.44	0.0002	0.01	collagen, type XXVII, alpha 1
COL27A1	225288_at	0.55	0.0000	0.01	collagen, type XXVII, alpha 1
COL4A5	213110_s_at	0.38	0.0004	0.01	collagen, type IV, alpha 5
COL4A6	213992_at	0.36	0.0008	0.02	collagen, type IV, alpha 6
COL7A1	204136_at	0.33	0.0002	0.01	collagen, type VII, alpha 1
CREBZF	225595_at	0.45	0.0005	0.02	CREB/ATF bZIP transcription factor
CREBZF	225594_at	0.48	0.0001	0.01	CREB/ATF bZIP transcription factor
CSNK1A1	1556006_s_at	0.46	0.0000	0.01	casein kinase 1, alpha 1
CXorf50B	242292_at	0.35	0.0001	0.01	non-protein coding RNA 246B
D4S234E	209570_s_at	0.34	0.0008	0.02	DNA segment on chromosome 4 (unique) 234 expressed sequence
DDX17	213998_s_at	0.49	0.0001	0.01	DEAD (Asp-Glu-Ala-Asp) box polypeptide 17
DDX26B	227485_at	0.36	0.0003	0.01	DEAD/H (Asp-Glu-Ala-Asp/His) box polypeptide 26B
DIDO1	213213_at	0.30	0.0007	0.02	death inducer-obliterator 1
DKFZp667E0512	236079_at	0.47	0.0003	0.01	hypothetical protein DKFZp667E0512
DNAH5	232381_s_at	0.36	0.0009	0.02	dynein, axonemal, heavy chain 5
DNALI1	227081_at	0.37	0.0001	0.01	dynein, axonemal, light intermediate chain 1
DOCK9	232874_at	-0.38	0.0001	0.01	dedicator of cytokinesis 9
DOPEY1	213267_at	0.35	0.0009	0.02	dopey family member 1
DSC3	206032_at	0.51	0.0000	0.01	desmocollin 3
DSC3	206033_s_at	0.55	0.0000	0.01	desmocollin 3
DYX1C1	235273_at	0.31	0.0000	0.01	dyslexia susceptibility 1 candidate 1
EBF1	232204_at	-0.33	0.0005	0.02	early B-cell factor 1
ECHDC2	235305_s_at	0.33	0.0009	0.02	enoyl CoA hydratase domain containing 2
EED	210656_at	0.35	0.0007	0.02	embryonic ectoderm development
EFHC1	219833_s_at	0.31	0.0001	0.01	EF-hand domain (C-terminal) containing 1
ELMO3	219411_at	0.30	0.0003	0.01	engulfment and cell motility 3
ENOSF1	213645_at	0.47	0.0001	0.01	enolase superfamily member 1
ENPP5	237054_at	0.37	0.0006	0.02	ectonucleotide pyrophosphatase/phosphodiesterase 5 (putative function)
EPM2AIP1	236314_at	0.48	0.0005	0.02	EPM2A (laforin) interacting protein 1
EZH2	203358_s_at	0.44	0.0000	0.01	enhancer of zeste homolog 2 (Drosophila)
FAM101A	227320_at	0.33	0.0000	0.00	family with sequence similarity 101, member A
FLJ25006	1553292_s_at	0.35	0.0008	0.02	uncharacterized serine/threonine-protein kinase SgK494
FLJ40330	1569040_s_at	0.73	0.0002	0.01	hypothetical LOC645784
FNBP4	212232_at	0.36	0.0003	0.01	formin binding protein 4
FRMD3	230645_at	-0.32	0.0005	0.02	FERM domain containing 3
FUBP1	214093_s_at	0.47	0.0002	0.01	far upstream element (FUSE) binding protein 1
GALNT6	219956_at	0.44	0.0007	0.02	UDP-N-acetyl-alpha-D-galactosamine:polypeptide N-acetylgalactosaminyltransferase 6 (GalNAc-T6)
GAS5	227517_s_at	0.70	0.0001	0.01	growth arrest-specific 5 (non-protein coding)
GATA3	209602_s_at	0.35	0.0009	0.02	GATA binding protein 3
GATA3	209603_at	0.38	0.0009	0.02	GATA binding protein 3
GOLGA2B	233198_at	0.32	0.0001	0.01	golgin A2 family, member B
GOLGA8A	208798_x_at	0.75	0.0002	0.01	golgin A8 family, member A
GOLGA8B	210425_x_at	0.61	0.0003	0.01	golgin A8 family, member B
HINT1	1555960_at	0.40	0.0002	0.01	histidine triad nucleotide binding protein 1
HNRNPA1	221919_at	0.50	0.0003	0.01	heterogeneous nuclear ribonucleoprotein A1
HNRNPA2B1	225932_s_at	0.32	0.0003	0.01	heterogeneous nuclear ribonucleoprotein

					A2/B1
HNRNPA2B1	225107_at	0.56	0.0003	0.01	heterogeneous nuclear ribonucleoprotein A2/B1
HNRNPD	213359_at	0.59	0.0003	0.01	heterogeneous nuclear ribonucleoprotein D (AU-rich element RNA binding protein 1, 37kDa)
HNRPDL	212454_x_at	0.65	0.0001	0.01	heterogeneous nuclear ribonucleoprotein D-like
HSPB11	214163_at	0.46	0.0002	0.01	heat shock protein family B (small), member 11
IGF1	209540_at	-0.35	0.0002	0.01	insulin-like growth factor 1 (somatomedin C)
IL28RA	244261_at	0.34	0.0003	0.01	interleukin 28 receptor, alpha (interferon, lambda receptor)
IL7R	226218_at	0.53	0.0001	0.01	interleukin 7 receptor
INTU	228946_at	0.51	0.0001	0.01	inturned planar cell polarity effector homolog (Drosophila)
IPW	221974_at	0.42	0.0001	0.01	imprinted in Prader-Willi syndrome (non-protein coding)
IQCA1	238584_at	0.50	0.0000	0.01	IQ motif containing with AAA domain 1
KCNE1	236407_at	0.46	0.0008	0.02	potassium voltage-gated channel, Isk-related family, member 1
KIAA0907	202220_at	0.33	0.0007	0.02	KIAA0907
KLHL29	229310_at	0.31	0.0000	0.01	kelch-like 29 (Drosophila)
KLK7	239381_at	0.44	0.0007	0.02	kallikrein-related peptidase 7
KRT15	204734_at	0.55	0.0001	0.01	keratin 15
KRT5	201820_at	0.41	0.0002	0.01	keratin 5
L3MBTL	210306_at	0.40	0.0001	0.01	l(3)mbt-like (Drosophila)
LAMA3	203726_s_at	0.30	0.0008	0.02	laminin, alpha 3
LAMC2	202267_at	0.34	0.0008	0.02	laminin, gamma 2
LNPEP	231866_at	-0.30	0.0001	0.01	leucyl/cystinyl aminopeptidase
LOC100127980	242462_at	0.30	0.0010	0.02	hypothetical protein LOC100127980
LOC100130360	243179_at	0.31	0.0008	0.02	hypothetical LOC100130360
LOC100286909	228528_at	0.41	0.0007	0.02	hypothetical protein LOC100286909
LOC150759	213703_at	0.55	0.0003	0.01	hypothetical protein LOC150759
LOC153682	232794_at	0.37	0.0001	0.01	hypothetical protein LOC153682
LOC221442	236832_at	0.40	0.0003	0.01	adenylate cyclase 10 pseudogene
LOC284513	1556597_a_at	0.38	0.0003	0.01	hypothetical protein LOC284513
LOC339047	221501_x_at	0.40	0.0001	0.01	hypothetical protein LOC339047
LOC399491	214035_x_at	0.36	0.0001	0.01	GPS, PLAT and transmembrane domain-containing protein
LOC642587	226755_at	0.64	0.0000	0.01	NPC-A-5
LUC7L3	220044_x_at	0.32	0.0001	0.01	LUC7-like 3 (S. cerevisiae)
LUC7L3	203804_s_at	0.34	0.0002	0.01	LUC7-like 3 (S. cerevisiae)
MALAT1	224558_s_at	0.56	0.0000	0.01	metastasis associated lung adenocarcinoma transcript 1 (non-protein coding)
MARCH3	213256_at	-0.30	0.0003	0.01	membrane-associated ring finger (C3HC4) 3
MBD4	214048_at	0.36	0.0003	0.01	methyl-CpG binding domain protein 4
MCTS1	230235_at	0.31	0.0004	0.01	malignant T cell amplified sequence 1
MDM1	213761_at	0.31	0.0001	0.01	Mdm1 nuclear protein homolog (mouse)
METTL3	213653_at	0.69	0.0000	0.01	methyltransferase like 3
MGP	238481_at	0.53	0.0003	0.01	matrix Gla protein
MPPED2	205413_at	0.39	0.0000	0.00	metallophosphoesterase domain containing 2
MREG	232682_at	0.35	0.0005	0.02	melanoregulin
MREG	219648_at	0.45	0.0001	0.01	melanoregulin
N4BP2L2	235547_at	0.33	0.0002	0.01	NEDD4 binding protein 2-like 2
NANOS1	228523_at	-0.38	0.0002	0.01	nanos homolog 1 (Drosophila)
NCRNA00173	237591_at	0.39	0.0002	0.01	non-protein coding RNA 173
NCRNA00201	225786_at	0.47	0.0004	0.01	non-protein coding RNA 201

NDUFB8	214241_at	0.31	0.0004	0.01	NADH dehydrogenase (ubiquinone) 1 beta subcomplex, 8, 19kDa
NFKBIZ	223217_s_at	0.42	0.0002	0.01	nuclear factor of kappa light polypeptide gene enhancer in B-cells inhibitor, zeta
NFKBIZ	223218_s_at	0.48	0.0001	0.01	nuclear factor of kappa light polypeptide gene enhancer in B-cells inhibitor, zeta
NKTR	202380_s_at	0.34	0.0009	0.02	natural killer-tumor recognition sequence
NLRC3	236295_s_at	0.32	0.0001	0.01	NLR family, CARD domain containing 3
NPHP3	235432_at	0.34	0.0006	0.02	nephronophthisis 3 (adolescent)
NPHP3	235410_at	0.38	0.0001	0.01	nephronophthisis 3 (adolescent)
NPIP	204538_x_at	0.38	0.0001	0.01	nuclear pore complex interacting protein
NRXN1	228547_at	0.60	0.0001	0.01	neurexin 1
NSMCE4A	228506_at	0.62	0.0002	0.01	non-SMC element 4 homolog A (S. cerevisiae)
NTF4	231785_at	0.36	0.0001	0.01	neurotrophin 4
OGT	207564_x_at	0.33	0.0001	0.01	O-linked N-acetylglucosamine (GlcNAc) transferase (UDP-N-acetylglucosamine:polypeptide-N-acetylglucosaminyl transferase)
OGT	212307_s_at	0.35	0.0001	0.01	O-linked N-acetylglucosamine (GlcNAc) transferase (UDP-N-acetylglucosamine:polypeptide-N-acetylglucosaminyl transferase)
OGT	229787_s_at	0.37	0.0001	0.01	O-linked N-acetylglucosamine (GlcNAc) transferase (UDP-N-acetylglucosamine:polypeptide-N-acetylglucosaminyl transferase)
OXTR	206825_at	0.54	0.0006	0.02	oxytocin receptor
PABPC1L	231838_at	0.37	0.0005	0.02	poly(A) binding protein, cytoplasmic 1-like
PABPN1	213046_at	0.35	0.0007	0.02	poly(A) binding protein, nuclear 1
PALM2	1554640_at	-0.32	0.0001	0.01	paralemmin 2
PDLIM4	211564_s_at	0.35	0.0002	0.01	PDZ and LIM domain 4
PDZD2	233025_at	-0.35	0.0004	0.01	PDZ domain containing 2
PIGL	232262_at	0.38	0.0005	0.02	phosphatidylinositol glycan anchor biosynthesis, class L
PILRB	220954_s_at	0.51	0.0000	0.01	paired immunoglobin-like type 2 receptor beta
PILRB	225321_s_at	0.64	0.0001	0.01	paired immunoglobin-like type 2 receptor beta
PNN	1567214_a_at	0.37	0.0003	0.01	pinin, desmosome associated protein
PNN	212036_s_at	0.37	0.0001	0.01	pinin, desmosome associated protein
PPM1K	235061_at	0.33	0.0004	0.01	protein phosphatase, Mg2+/Mn2+ dependent, 1K
PRPF39	220553_s_at	0.31	0.0000	0.01	PRP39 pre-mRNA processing factor 39 homolog (S. cerevisiae)
PRPF4B	202127_at	0.44	0.0005	0.02	PRP4 pre-mRNA processing factor 4 homolog B (yeast)
PTN	209465_x_at	0.55	0.0005	0.02	pleiotrophin
PTN	211737_x_at	0.66	0.0002	0.01	pleiotrophin
PTN	209466_x_at	0.67	0.0002	0.01	pleiotrophin
RALGAPA2	232500_at	-0.30	0.0002	0.01	Ral GTPase activating protein, alpha subunit 2 (catalytic)
RASD1	223467_at	-0.31	0.0009	0.02	RAS, dexamethasone-induced 1
RBBP8	203344_s_at	0.32	0.0000	0.01	retinoblastoma binding protein 8
RBM25	1557081_at	0.38	0.0009	0.02	RNA binding motif protein 25
RBMX	225310_at	0.38	0.0000	0.01	RNA binding motif protein, X-linked
RDH13	1559190_s_at	0.35	0.0001	0.01	retinol dehydrogenase 13 (all-trans/9-cis)
RGS1	216834_at	0.45	0.0005	0.02	regulator of G-protein signaling 1
RNF183	235153_at	0.49	0.0001	0.01	ring finger protein 183
RPL10	221989_at	0.59	0.0000	0.01	ribosomal protein L10
RPL37A	213459_at	0.50	0.0004	0.01	ribosomal protein L37a

RPS24	1555878_at	0.55	0.0004	0.02	ribosomal protein S24
RUNX3	204198_s_at	0.31	0.0001	0.01	runt-related transcription factor 3
RUNX3	204197_s_at	0.36	0.0000	0.01	runt-related transcription factor 3
RYR3	206306_at	0.40	0.0003	0.01	ryanodine receptor 3
SCARNA15	228930_at	0.33	0.0000	0.01	small Cajal body-specific RNA 15
SCNN1A	203453_at	0.40	0.0003	0.01	sodium channel, nonvoltage-gated 1 alpha
SCUBE2	219197_s_at	0.42	0.0009	0.02	signal peptide, CUB domain, EGF-like 2
SFI1	36545_s_at	0.32	0.0000	0.01	Sfi1 homolog, spindle assembly associated (yeast)
SFPQ	214016_s_at	0.46	0.0002	0.01	splicing factor proline/glutamine-rich
SFPQ	221768_at	0.47	0.0006	0.02	splicing factor proline/glutamine-rich
SFRS1	201742_x_at	0.30	0.0001	0.01	splicing factor, arginine/serine-rich 1
SFRS18	212179_at	0.39	0.0003	0.01	splicing factor, arginine/serine-rich 18
SFRS18	212176_at	0.39	0.0002	0.01	splicing factor, arginine/serine-rich 18
SFRS18	212177_at	0.40	0.0002	0.01	splicing factor, arginine/serine-rich 18
SFRS5	203380_x_at	0.31	0.0001	0.01	splicing factor, arginine/serine-rich 5
SFRS5	212266_s_at	0.36	0.0000	0.01	splicing factor, arginine/serine-rich 5
SFRS7	213649_at	0.40	0.0004	0.01	splicing factor, arginine/serine-rich 7, 35kDa
SGSM2	217538_at	0.50	0.0001	0.01	small G protein signaling modulator 2
SLC16A6	230748_at	0.38	0.0009	0.02	solute carrier family 16, member 6 (monocarboxylic acid transporter 7)
SLC25A27	1554161_at	0.36	0.0008	0.02	solute carrier family 25, member 27
SLC25A27	230624_at	0.55	0.0006	0.02	solute carrier family 25, member 27
SLC27A6	219932_at	0.48	0.0005	0.02	solute carrier family 27 (fatty acid transporter), member 6
SLC4A7	209884_s_at	0.31	0.0001	0.01	solute carrier family 4, sodium bicarbonate cotransporter, member 7
SLFN13	1553423_a_at	0.31	0.0002	0.01	schlafen family member 13
SNHG10	244786_at	0.62	0.0000	0.01	small nucleolar RNA host gene 10 (non-protein coding)
SNHG12	228990_at	0.43	0.0000	0.01	small nucleolar RNA host gene 12 (non-protein coding)
SNHG12	223773_s_at	0.53	0.0000	0.01	small nucleolar RNA host gene 12 (non-protein coding)
SNORD104	228879_at	0.43	0.0000	0.01	small nucleolar RNA, C/D box 104
SOX6	227498_at	-0.33	0.0007	0.02	SRY (sex determining region Y)-box 6
SOX6	228214_at	-0.31	0.0000	0.01	SRY (sex determining region Y)-box 6
STC2	203439_s_at	0.49	0.0008	0.02	stanniocalcin 2
SYCP2	206546_at	0.45	0.0000	0.01	synaptonemal complex protein 2
SYTL1	227134_at	0.37	0.0005	0.02	synaptotagmin-like 1
TCTE3	1557945_at	0.35	0.0004	0.01	t-complex-associated-testis-expressed 3
TNMD	220065_at	-0.58	0.0009	0.02	tenomodulin
TRAF3IP3	213888_s_at	0.31	0.0006	0.02	TRAF3 interacting protein 3
TTC14	225180_at	0.37	0.0002	0.01	tetratricopeptide repeat domain 14
TTC18	229170_s_at	0.41	0.0002	0.01	tetratricopeptide repeat domain 18
TTC6	1556666_a_at	0.76	0.0001	0.01	tetratricopeptide repeat domain 6
UQCC	229672_at	0.35	0.0001	0.01	ubiquinol-cytochrome c reductase complex chaperone
UROD	222074_at	0.33	0.0000	0.01	uroporphyrinogen decarboxylase
VAMP1	213326_at	0.40	0.0000	0.01	vesicle-associated membrane protein 1 (synaptobrevin 1)
WDR52	230152_at	0.39	0.0005	0.02	WD repeat domain 52
WDR90	227894_at	0.32	0.0000	0.01	WD repeat domain 90
WSB1	210561_s_at	0.35	0.0003	0.01	WD repeat and SOCS box-containing 1
XIST	224589_at	0.39	0.0000	0.01	X (inactive)-specific transcript (non-protein coding)
XIST	221728_x_at	0.57	0.0000	0.01	X (inactive)-specific transcript (non-protein coding)

XIST	214218_s_at	0.57	0.0000	0.01	X (inactive)-specific transcript (non-protein coding)
ZDHHC11	1552283_s_at	0.37	0.0000	0.01	zinc finger, DHHC-type containing 11
ZDHHC11	221646_s_at	0.42	0.0000	0.01	zinc finger, DHHC-type containing 11
ZMAT1	226344_at	0.47	0.0005	0.02	zinc finger, matrin type 1
ZNF107	205739_x_at	0.37	0.0001	0.01	zinc finger protein 107
ZNF207	1556035_s_at	0.41	0.0007	0.02	zinc finger protein 207
ZNF207	228157_at	0.59	0.0002	0.01	zinc finger protein 207
ZNF692	220661_s_at	0.34	0.0000	0.00	zinc finger protein 692
ZNF711	228988_at	0.41	0.0003	0.01	zinc finger protein 711
ZNF767	219627_at	0.41	0.0002	0.01	zinc finger family member 767
ZNF785	1554769_at	0.32	0.0003	0.01	zinc finger protein 785
ZNF789	235231_at	0.32	0.0005	0.02	zinc finger protein 789
ZNF814	60794_f_at	0.34	0.0006	0.02	zinc finger protein 814
ZNF83	221645_s_at	0.42	0.0001	0.01	zinc finger protein 83
	215768_at	-0.49	0.0003	0.01	
	244876_at	-0.47	0.0000	0.01	
	1556935_at	-0.44	0.0004	0.02	
	233648_at	-0.44	0.0006	0.02	
	242736_at	-0.43	0.0002	0.01	
	1560049_at	-0.43	0.0000	0.01	
	1568920_at	-0.42	0.0001	0.01	
	1570246_at	-0.42	0.0006	0.02	
	239220_at	-0.42	0.0008	0.02	
	232331_at	-0.40	0.0009	0.02	
	242868_at	-0.40	0.0000	0.01	
	240772_at	-0.38	0.0000	0.01	
	232882_at	-0.37	0.0003	0.01	
	231644_at	-0.35	0.0009	0.02	
	228731_at	-0.33	0.0008	0.02	
	243819_at	-0.33	0.0008	0.02	
	234224_at	-0.33	0.0003	0.01	
	234074_at	-0.33	0.0001	0.01	
	243039_at	-0.31	0.0001	0.01	
	232582_at	-0.30	0.0002	0.01	
	239102_s_at	-0.30	0.0005	0.02	
	232852_at	-0.30	0.0007	0.02	
	230664_at	0.31	0.0002	0.01	
	238466_at	0.32	0.0001	0.01	
	230314_at	0.32	0.0001	0.01	
	228539_at	0.32	0.0001	0.01	
	229654_at	0.32	0.0005	0.02	
	223528_s_at	0.32	0.0000	0.01	
	229830_at	0.33	0.0003	0.01	
	229926_at	0.33	0.0004	0.01	
	1557383_a_at	0.35	0.0003	0.01	
	239624_at	0.35	0.0007	0.02	
	235611_at	0.36	0.0003	0.01	
	239644_at	0.36	0.0002	0.01	
	244663_at	0.37	0.0002	0.01	
	226316_at	0.37	0.0002	0.01	
	239209_at	0.38	0.0001	0.01	
	244271_at	0.38	0.0003	0.01	
	214870_x_at	0.38	0.0001	0.01	
	228464_at	0.39	0.0002	0.01	
	230304_at	0.39	0.0001	0.01	

229111_at	0.39	0.0003	0.01
235892_at	0.40	0.0001	0.01
213212_x_at	0.41	0.0006	0.02
235607_at	0.41	0.0001	0.01
222018_at	0.41	0.0008	0.02
214291_at	0.42	0.0005	0.02
235535_x_at	0.43	0.0008	0.02
238501_at	0.44	0.0010	0.02
240405_at	0.44	0.0001	0.01
240182_at	0.45	0.0000	0.01
226444_at	0.45	0.0010	0.02
214683_s_at	0.48	0.0004	0.01
228465_at	0.51	0.0001	0.01
221973_at	0.55	0.0001	0.01
241863_x_at	0.55	0.0000	0.01
229455_at	0.55	0.0001	0.01
235771_at	0.55	0.0001	0.01
230333_at	0.59	0.0003	0.01
235902_at	0.60	0.0001	0.01
1556839_s_at	0.62	0.0000	0.01
229795_at	0.64	0.0001	0.01

Figure S1

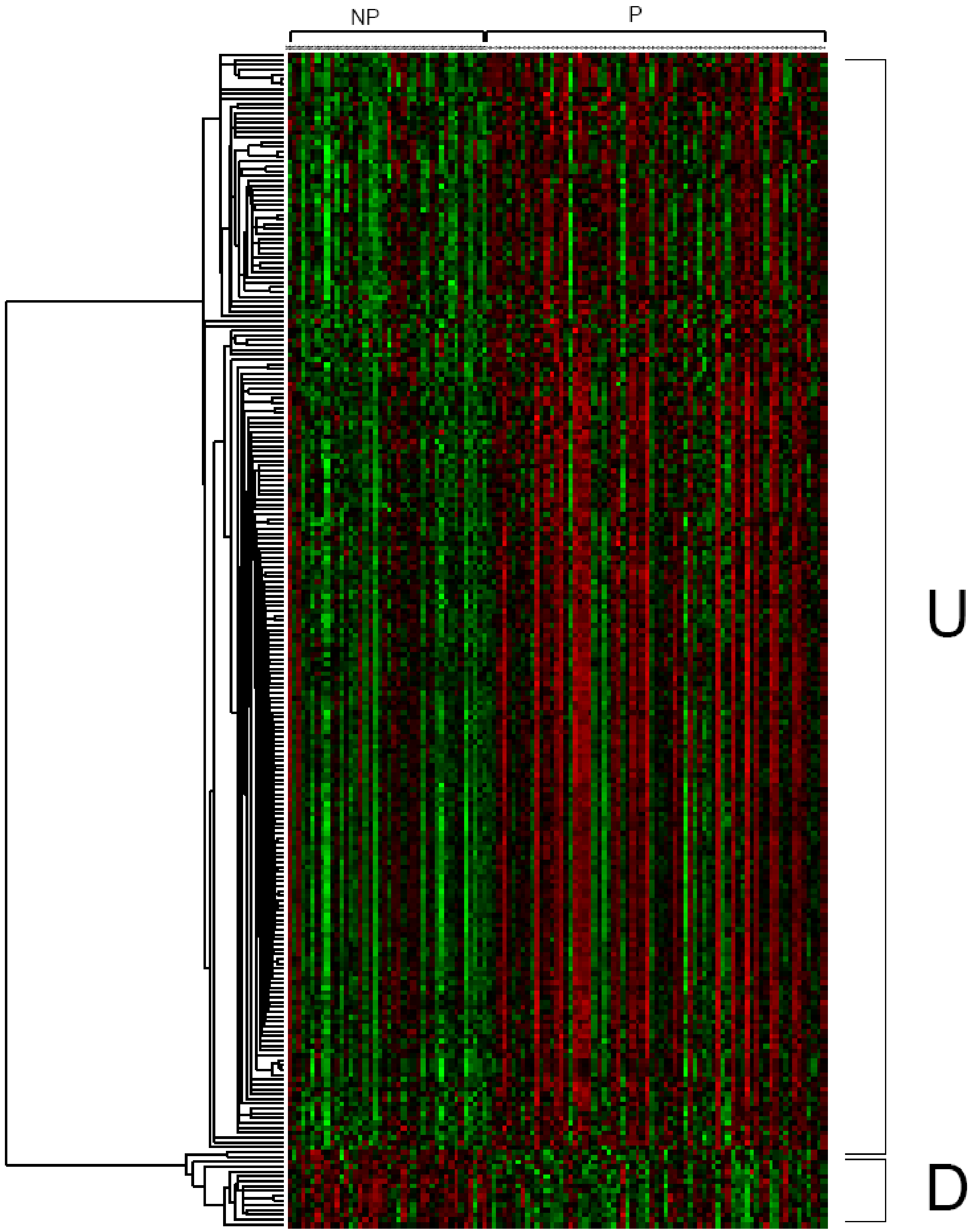

Figure S1- Hierarchical clustering of differentially expressed probesets in parous and nulliparous women (samples were not clustered). Red represents expression values above the median across all samples, and green represents values below the median. The left portion of the figure is composed by nulliparous (NP) samples and the right portion is composed by parous (P) samples. 'U' represents the intensity of up-regulated probesets among parous samples whereas 'D' represents the intensity of down-regulated probesets.

Table S2- Genes differentially expressed by full term pregnancy (P) when compared to women that did not have a full term pregnancy (GN) (p<0.001 and log_2 fold change of at least 0.3)

Gene Symbol	Gene Name	Probe ID	Log Ratio	P value	FDR
ADCYAP1R1	adenylate cyclase activating polypeptide 1 (pituitary) receptor type I	226690_at	-0.35	0.0005	0.41
BMPR1B	bone morphogenetic protein receptor, type IB	229975_at	1.17	0.0001	0.41
C8orf84	chromosome 8 open reading frame 84	235209_at	0.67	0.0007	0.41
C8orf84	chromosome 8 open reading frame 84	235210_s_at	0.68	0.0009	0.41
C9orf5	chromosome 9 open reading frame 5	223007_s_at	-0.31	0.0005	0.41
CDK5RAP3	CDK5 regulatory subunit associated protein 3	218740_s_at	0.34	0.0007	0.41
CLN5	ceroid-lipofuscinosis, neuronal 5	214252_s_at	0.33	0.0001	0.41
CYTSB	cytospin B	228900_at	-0.35	0.0009	0.41
FAM101A	family with sequence similarity 101, member A	227320_at	0.39	0.0007	0.41
MIA	melanoma inhibitory activity	206560_s_at	0.66	0.0008	0.41
MPPED2	metallophosphoesterase domain containing 2	205413_at	0.53	0.0001	0.41
SPRY4	sprouty homolog 4 (Drosophila)	221489_s_at	-0.38	0.0005	0.41
XIST	X (inactive)-specific transcript (non-protein coding)	224589_at	0.53	0.0005	0.41
		236373_at	-0.42	0.0001	0.41
		237190_at	-0.32	0.0002	0.41
		232626_at	0.35	0.0010	0.41
		235762_at	0.39	0.0010	0.41
		241600_at	0.40	0.0009	0.41

Table S3. Genes differentially expressed in Parous (P) versus Nulligravidas (NG) ($p<0.001$ and log_2 fold change of at least 0.3)

Gene Symbol	Gene Name	Probe ID	Log Ratio	P value	Adj.P. value
ABAT	4-aminobutyrate aminotransferase	209459_s_at	0.35	0.0005	0.03
ABHD5	abhydrolase domain containing 5	213805_at	-0.33	0.0009	0.03
AGBL2	ATP/GTP binding protein-like 2	220390_at	0.32	0.0001	0.02
ANKRD10	ankyrin repeat domain 10	223251_s_at	0.31	0.0008	0.03
ANXA9	annexin A9	211712_s_at	0.41	0.0004	0.03
AP1G2	adaptor-related protein complex 1, gamma 2 subunit	201613_s_at	0.37	0.0002	0.02
ARGLU1	arginine and glutamate rich 1	228477_at	0.33	0.0005	0.03
BZRAP1	benzodiazapine receptor (peripheral) associated protein 1	205839_s_at	0.31	0.0004	0.03
C11orf31	chromosome 11 open reading frame 31	228331_at	0.47	0.0004	0.03
C1orf168	chromosome 1 open reading frame 168	238625_at	0.82	0.0000	0.02
CAPN8	calpain 8	229030_at	0.61	0.0001	0.02
CCNL1	cyclin L1	220046_s_at	0.42	0.0003	0.03
CCNL2	cyclin L2	222999_s_at	0.47	0.0009	0.03
CDK12	cyclin-dependent kinase 12	213557_at	0.41	0.0001	0.02
CELF2	CUGBP, Elav-like family member 2	242268_at	-0.36	0.0004	0.03
CGN	cingulin	223233_s_at	0.41	0.0003	0.03
CGN	cingulin	223232_s_at	0.46	0.0002	0.02
CHD2	chromodomain helicase DNA binding protein 2	228999_at	0.31	0.0006	0.03
CIRBP	cold inducible RNA binding protein	230142_s_at	0.41	0.0003	0.03
CIRBP	cold inducible RNA binding protein	225191_at	0.57	0.0005	0.03
CMYA5	cardiomyopathy associated 5	233520_s_at	0.50	0.0000	0.01
COL27A1	collagen, type XXVII, alpha 1	225288_at	0.55	0.0002	0.02
CREBZF	CREB/ATF bZIP transcription factor	225594_at	0.47	0.0008	0.03
CSNK1A1	casein kinase 1, alpha 1	1556006_s_at	0.44	0.0003	0.03
CXorf50B	chromosome X open reading frame 50B	242292_at	0.34	0.0009	0.04
DLC1	deleted in liver cancer 1	220512_at	-0.35	0.0008	0.03
DNAH5	dynein, axonemal, heavy chain 5	232381_s_at	0.42	0.0005	0.03
DNALI1	dynein, axonemal, light intermediate chain 1	227081_at	0.35	0.0006	0.03
DOCK9	dedicator of cytokinesis 9	232874_at	-0.45	0.0000	0.01
DPP7	dipeptidyl-peptidase 7	238012_at	0.31	0.0001	0.02
DSC3	desmocollin 3	206032_at	0.47	0.0007	0.03
DSC3	desmocollin 3	206033_s_at	0.55	0.0000	0.02
DYX1C1	dyslexia susceptibility 1 candidate 1	235273_at	0.34	0.0000	0.02
EBF1	early B-cell factor 1	233261_at	-0.50	0.0007	0.03
ELMO3	engulfment and cell motility 3	219411_at	0.31	0.0006	0.03
ENOSF1	enolase superfamily member 1	213645_at	0.50	0.0002	0.02
EPS8L2	EPS8-like 2	218180_s_at	0.32	0.0002	0.02
EZH2	enhancer of zeste homolog 2 (Drosophila)	203358_s_at	0.42	0.0002	0.02
FAM101A	family with sequence similarity 101, member A	227320_at	0.31	0.0001	0.02
FNBP4	formin binding protein 4	212232_at	0.37	0.0009	0.03
FYB	FYN binding protein (FYB-120/130)	227266_s_at	0.33	0.0002	0.02

GALNT6	UDP-N-acetyl-alpha-D-galactosamine:polypeptide N-acetylgalactosaminyltransferase 6 (GalNAc-T6)	219956_at	0.49	0.0006	0.03
GAS5	growth arrest-specific 5 (non-protein coding)	227517_s_at	0.70	0.0003	0.03
GATA3	GATA binding protein 3	209602_s_at	0.39	0.0009	0.03
GATA3	GATA binding protein 3	209603_at	0.44	0.0005	0.03
GOLGA2B	golgin A2 family, member B	233198_at	0.31	0.0006	0.03
GOLGA8A	golgin A8 family, member A	208798_x_at	0.77	0.0007	0.03
HINT1	histidine triad nucleotide binding protein 1	1555960_at	0.41	0.0006	0.03
HNRPDL	heterogeneous nuclear ribonucleoprotein D-like	212454_x_at	0.65	0.0004	0.03
IGF1	insulin-like growth factor 1 (somatomedin C)	209540_at	-0.37	0.0004	0.03
IL7R	interleukin 7 receptor	226218_at	0.56	0.0002	0.02
INTU	inturned planar cell polarity effector homolog (Drosophila)	228946_at	0.53	0.0005	0.03
IPW	imprinted in Prader-Willi syndrome (non-protein coding)	221974_at	0.43	0.0002	0.02
IQCA1	IQ motif containing with AAA domain 1	238584_at	0.46	0.0001	0.02
KRT5	keratin 5	201820_at	0.41	0.0010	0.04
L3MBTL	l(3)mbt-like (Drosophila)	210306_at	0.37	0.0010	0.04
LAMC2	laminin, gamma 2	202267_at	0.38	0.0008	0.03
LNPEP	leucyl/cystinyl aminopeptidase	231866_at	-0.31	0.0003	0.03
LOC100286909	hypothetical protein LOC100286909	228528_at	0.45	0.0009	0.03
LOC153682	hypothetical protein LOC153682	232794_at	0.37	0.0004	0.03
LOC220594	TL132 protein	213510_x_at	0.30	0.0006	0.03
LOC221442	adenylate cyclase 10 pseudogene	236832_at	0.42	0.0007	0.03
LOC284513	hypothetical protein LOC284513	1556597_a_at	0.40	0.0009	0.03
LOC339047	hypothetical protein LOC339047	221501_x_at	0.45	0.0001	0.02
LOC399491	GPS, PLAT and transmembrane domain-containing protein	214035_x_at	0.39	0.0001	0.02
LOC642587	NPC-A-5	226755_at	0.64	0.0000	0.02
LUC7L3	LUC7-like 3 (S. cerevisiae)	220044_x_at	0.32	0.0005	0.03
LUC7L3	LUC7-like 3 (S. cerevisiae)	203804_s_at	0.33	0.0010	0.04
MALAT1	metastasis associated lung adenocarcinoma transcript 1 (non-protein coding)	224558_s_at	0.57	0.0001	0.02
MARCH3	membrane-associated ring finger (C3HC4) 3	213256_at	-0.36	0.0001	0.02
MDM1	Mdm1 nuclear protein homolog (mouse)	213761_at	0.31	0.0004	0.03
METTL3	methyltransferase like 3	213653_at	0.66	0.0001	0.02
MGC24103	hypothetical MGC24103	232568_at	-0.38	0.0005	0.03
MPPED2	metallophosphoesterase domain containing 2	205413_at	0.35	0.0001	0.02
MREG	melanoregulin	219648_at	0.48	0.0001	0.02
MRI1	methylthioribose-1-phosphate isomerase homolog (S. cerevisiae)	228077_at	0.30	0.0009	0.03
NDUFB8	NADH dehydrogenase (ubiquinone) 1 beta subcomplex, 8, 19kDa	214241_at	0.34	0.0005	0.03
NFKBIZ	nuclear factor of kappa light polypeptide gene enhancer in B-cells inhibitor, zeta	223217_s_at	0.45	0.0003	0.03

NFKBIZ	nuclear factor of kappa light polypeptide gene enhancer in B-cells inhibitor, zeta	223218_s_at	0.50	0.0002	0.02
NINL	ninein-like	207705_s_at	0.30	0.0000	0.02
NLRC3	NLR family, CARD domain containing 3	236295_s_at	0.35	0.0001	0.02
NPHP3	nephronophthisis 3 (adolescent)	235410_at	0.37	0.0005	0.03
NPIP	nuclear pore complex interacting protein	204538_x_at	0.42	0.0001	0.02
NRXN1	neurexin 1	228547_at	0.58	0.0008	0.03
OGT	O-linked N-acetylglucosamine (GlcNAc) transferase (UDP-N-acetylglucosamine:polypeptide-N-acetylglucosaminyl transferase)	207564_x_at	0.33	0.0008	0.03
OGT	O-linked N-acetylglucosamine (GlcNAc) transferase (UDP-N-acetylglucosamine:polypeptide-N-acetylglucosaminyl transferase)	212307_s_at	0.36	0.0005	0.03
OGT	O-linked N-acetylglucosamine (GlcNAc) transferase (UDP-N-acetylglucosamine:polypeptide-N-acetylglucosaminyl transferase)	229787_s_at	0.36	0.0007	0.03
OXTR	oxytocin receptor	206825_at	0.63	0.0003	0.03
PALM2	paralemmin 2	1554640_at	-0.37	0.0001	0.02
PAN2	PAN2 poly(A) specific ribonuclease subunit homolog (S. cerevisiae)	203117_s_at	0.31	0.0004	0.03
PDZD2	PDZ domain containing 2	233025_at	-0.41	0.0002	0.02
PILRB	paired immunoglobin-like type 2 receptor beta	220954_s_at	0.51	0.0001	0.02
PILRB	paired immunoglobin-like type 2 receptor beta	225321_s_at	0.66	0.0004	0.03
PNN	pinin, desmosome associated protein	212036_s_at	0.36	0.0008	0.03
PPP1R16B	protein phosphatase 1, regulatory (inhibitor) subunit 16B	233813_at	-0.33	0.0008	0.03
RALGAPA2	Ral GTPase activating protein, alpha subunit 2 (catalytic)	232500_at	-0.38	0.0000	0.02
RASAL2	RAS protein activator like 2	1557432_at	-0.45	0.0003	0.03
RBBP8	retinoblastoma binding protein 8	203344_s_at	0.35	0.0000	0.01
RBMX	RNA binding motif protein, X-linked	225310_at	0.38	0.0001	0.02
RDH13	retinol dehydrogenase 13 (all-trans/9-cis)	1559190_s_at	0.39	0.0001	0.02
RPL10	ribosomal protein L10	221989_at	0.59	0.0003	0.03
RUNX3	runt-related transcription factor 3	204198_s_at	0.32	0.0004	0.03
RUNX3	runt-related transcription factor 3	204197_s_at	0.36	0.0001	0.02
RYR3	ryanodine receptor 3	206306_at	0.41	0.0009	0.03
SCARNA15	small Cajal body-specific RNA 15	228930_at	0.31	0.0004	0.03
SCNN1A	sodium channel, nonvoltage-gated 1 alpha	203453_at	0.43	0.0005	0.03
SFI1	Sfi1 homolog, spindle assembly associated (yeast)	36545_s_at	0.35	0.0000	0.01
SFRS18	splicing factor, arginine/serine-rich 18	212176_at	0.40	0.0007	0.03
SFRS5	splicing factor, arginine/serine-rich 5	212266_s_at	0.33	0.0006	0.03
SLC16A1	solute carrier family 16, member 1 (monocarboxylic acid transporter 1)	202234_s_at	-0.31	0.0004	0.03

SLC4A7	solute carrier family 4, sodium bicarbonate cotransporter, member 7	209884_s_at	0.31	0.0003	0.03
SLFN13	schlafen family member 13	1553423_a_at	0.32	0.0009	0.04
SNHG10	small nucleolar RNA host gene 10 (non-protein coding)	244786_at	0.59	0.0005	0.03
SNHG12	small nucleolar RNA host gene 12 (non-protein coding)	228990_at	0.38	0.0009	0.03
SNHG12	small nucleolar RNA host gene 12 (non-protein coding)	223773_s_at	0.50	0.0001	0.02
SNORD104	small nucleolar RNA, C/D box 104	228879_at	0.41	0.0002	0.02
SNRK	SNF related kinase	207474_at	-0.32	0.0006	0.03
SOX6	SRY (sex determining region Y)-box 6	228214_at	-0.31	0.0001	0.02
SYCP2	synaptonemal complex protein 2	206546_at	0.49	0.0000	0.01
TGFBR1	transforming growth factor, beta receptor 1	236561_at	-0.31	0.0007	0.03
THSD7A	thrombospondin, type I, domain containing 7A	213894_at	-0.32	0.0004	0.03
TTC14	tetratricopeptide repeat domain 14	225180_at	0.37	0.0007	0.03
TTC18	tetratricopeptide repeat domain 18	229170_s_at	0.42	0.0007	0.03
TTC6	tetratricopeptide repeat domain 6	1556666_a_at	0.82	0.0002	0.02
UQCC	ubiquinol-cytochrome c reductase complex chaperone	229672_at	0.35	0.0005	0.03
VAMP1	vesicle-associated membrane protein 1 (synaptobrevin 1)	213326_at	0.41	0.0002	0.02
WDR90	WD repeat domain 90	227894_at	0.34	0.0001	0.02
XIST	X (inactive)-specific transcript (non-protein coding)	214218_s_at	0.57	0.0003	0.03
XIST	X (inactive)-specific transcript (non-protein coding)	221728_x_at	0.58	0.0001	0.02
ZDHHC11	zinc finger, DHHC-type containing 11	1552283_s_at	0.38	0.0001	0.02
ZDHHC11	zinc finger, DHHC-type containing 11	221646_s_at	0.45	0.0001	0.02
ZNF107	zinc finger protein 107	205739_x_at	0.40	0.0001	0.02
ZNF207	zinc finger protein 207	228157_at	0.59	0.0007	0.03
ZNF432	zinc finger protein 432	219848_s_at	0.32	0.0001	0.02
ZNF692	zinc finger protein 692	220661_s_at	0.34	0.0000	0.01
ZNF767	zinc finger family member 767	219627_at	0.42	0.0007	0.03
ZNF814	zinc finger protein 814	60794_f_at	0.37	0.0009	0.03
ZNF83	zinc finger protein 83	221645_s_at	0.40	0.0009	0.03
		215768_at	-0.64	0.0000	0.01
		233648_at	-0.54	0.0001	0.02
		244876_at	-0.54	0.0000	0.01
		1568920_at	-0.52	0.0000	0.01
		242736_at	-0.51	0.0001	0.02
		240772_at	-0.50	0.0000	0.01
		1560049_at	-0.50	0.0000	0.01
		1556935_at	-0.49	0.0005	0.03
		242868_at	-0.45	0.0000	0.01
		232882_at	-0.45	0.0001	0.02
		234224_at	-0.45	0.0000	0.01
		231644_at	-0.43	0.0002	0.02
		239519_at	-0.43	0.0010	0.04
		1556590_s_at	-0.42	0.0002	0.02
		215791_at	-0.42	0.0004	0.03

1558410_s_at	-0.41	0.0007	0.03
234074_at	-0.40	0.0000	0.01
239102_s_at	-0.40	0.0000	0.02
241681_at	-0.40	0.0001	0.02
242457_at	-0.38	0.0005	0.03
234563_at	-0.38	0.0001	0.02
1566825_at	-0.38	0.0000	0.01
1563467_at	-0.38	0.0004	0.03
232852_at	-0.38	0.0001	0.02
243819_at	-0.37	0.0006	0.03
1556658_a_at	-0.36	0.0007	0.03
242025_at	-0.36	0.0000	0.02
233689_at	-0.35	0.0000	0.02
237778_at	-0.34	0.0002	0.02
232582_at	-0.34	0.0002	0.02
242188_at	-0.34	0.0002	0.02
239809_at	-0.34	0.0004	0.03
242699_at	-0.34	0.0000	0.01
243039_at	-0.33	0.0002	0.02
236355_s_at	-0.33	0.0002	0.02
233455_at	-0.33	0.0007	0.03
234118_at	-0.33	0.0000	0.01
234033_at	-0.33	0.0006	0.03
236395_at	-0.33	0.0006	0.03
1556989_at	-0.32	0.0001	0.02
232776_at	-0.32	0.0006	0.03
239661_at	-0.32	0.0006	0.03
1566482_at	-0.31	0.0004	0.03
241435_at	-0.31	0.0000	0.02
242664_at	-0.31	0.0005	0.03
232472_at	-0.30	0.0007	0.03
244387_at	-0.30	0.0004	0.03
230314_at	0.31	0.0006	0.03
228539_at	0.32	0.0008	0.03
223528_s_at	0.33	0.0001	0.02
230664_at	0.33	0.0003	0.03
229654_at	0.35	0.0007	0.03
238466_at	0.35	0.0000	0.02
239644_at	0.37	0.0007	0.03
226316_at	0.38	0.0007	0.03
230304_at	0.38	0.0005	0.03
239209_at	0.39	0.0003	0.03
244663_at	0.39	0.0004	0.03
235607_at	0.40	0.0005	0.03
240182_at	0.42	0.0005	0.03
214870_x_at	0.42	0.0002	0.02
228465_at	0.50	0.0006	0.03
241863_x_at	0.54	0.0002	0.02
221973_at	0.54	0.0004	0.03
235771_at	0.55	0.0004	0.03
229455_at	0.55	0.0004	0.03
235902_at	0.58	0.0004	0.03
1556839_s_at	0.62	0.0002	0.02

Table S4. Comparison between biological processes that are over-represented in the two studies

	**Biological processes over-represented in different studies**	
	**Previous study (Russo el al. 2008)**	**Current study**
**RNA processing**	*GO:0006376- mRNA splice site selection*<br>*GO:0000398- nuclear mRNA splicing, via spliceosome*<br>*GO:0000375- RNA splicing, via transesterification reactions*<br>*GO:0000381- regulation of alternative nuclear mRNA splicing, via spliceosome*<br>GO:0006403- RNA localization	*GO:0006376- mRNA splice site selection*<br>*GO:0000398- nuclear mRNA splicing, via spliceosome*<br>*GO:0000375- RNA splicing, via transesterification reactions*<br>*GO:0000380- alternative nuclear mRNA splicing, via spliceosome*<br>GO:0006396- RNA processing<br>GO:0016070- RNA metabolic process
**Other processes**	GO:0007050- cell cycle arrest<br>GO:0016477- cell migration<br>GO:0006613- cotranslational protein targeting to membrane<br>GO:0048523- negative regulation of cellular process<br>GO:0006470- protein amino acid dephosphorylation<br>GO:0030150- protein import into mitochondrial matrix<br>GO:0006195- purine nucleotide catabolic process	GO:0007044- cell-substrate junction assembly<br>GO:0007398- ectoderm development<br>GO:0008544- epidermis development<br>GO:0010467- gene expression
